# Transcutaneous auricular vagus nerve stimulation may improve cognitive deficits in neuropsychiatric diseases—a systematic review

**DOI:** 10.3389/fnagi.2026.1735787

**Published:** 2026-02-04

**Authors:** Stefanie Theresa Jost, Fabienne Happe, Julian Koenig, Haidar S. Dafsari

**Affiliations:** 1 Department of Neurology, University of Cologne, Faculty of Medicine and University Hospital Cologne, Cologne, Germany; 2 Department of Child and Adolescent Psychiatry, Psychosomatics and Psychotherapy, Faculty of Medicine and University Hospital Cologne, University of Cologne, Cologne, Germany

**Keywords:** cognitive impairment, neuropsychiatric disorders, Parkinson’s disease, systematic review, transcutaneous auricular vagus nerve stimulation

## Abstract

**Background:**

Transcutaneous auricular vagus nerve stimulation (taVNS) is a safe, effective, and non-invasive therapeutic approach for various neuropsychiatric disorders, including depression, headache disorders, and epilepsy. Cognitive impairment is a common and clinically relevant feature across these conditions, often contributing to poor functional outcomes. While improvements in cognitive performance have been reported in healthy individuals undergoing taVNS, it remains unclear whether taVNS can also alleviate cognitive deficits in individuals with neuropsychiatric disorders.

**Methods:**

A comprehensive literature search was performed in PubMed, Embase, and the Cochrane Central Register of Controlled Trials, complemented by manual searches. Predefined inclusion and exclusion criteria were applied. Study selection and data extraction were conducted using the rayyan.ai platform. Reporting followed the Preferred Reporting Items for Systematic Reviews and Meta-Analyses (PRISMA) and Synthesis Without Meta-analysis (SWiM) guidelines. The methodological quality of included studies was assessed using the Cochrane Risk-of-Bias tool for randomized trials and the ROBINS-I tool for non-randomized studies. Extracted data included population, intervention, comparator, and clinical outcome variables, as well as stimulation parameters according to the international consensus on vagus nerve stimulation research. Cognitive domains investigated in each study were categorized, and results were summarized using mode statistics and range.

**Results:**

Out of 418 records identified, 146 duplicates were removed. Of the remaining 272 studies, 67 were excluded after title and abstract screening and 192 after full-text assessment. Two additional studies were identified through manual reference screening, resulting in a total of 15 included studies. Eight of these reported improvements in global cognition, attention, memory, language, executive functions or social cognition following taVNS.

**Discussion:**

Evidence from the included studies suggests that taVNS may improve cognitive performance in neuropsychiatric disorders. The underlying mechanisms are likely multifactorial, including localized effects within the brainstem and modulation of broader neural networks. Future studies with longer follow-up periods and standardized stimulation protocols are warranted to clarify the cognitive effects of taVNS in neuropsychiatric populations.

## 1 Introduction

Cognitive impairment is common across a wide range of neuropsychiatric disorders such as epilepsy, major depressive disorder, and Parkinson’s disease and substantially contributes to reduce daily functioning and quality of life (Millan et al., 2012; Litvan et al., 2012; Bora and Meletti, 2016). Effective treatments specifically targeting these cognitive symptoms remain limited, underscoring the need for novel neuromodulatory approaches.

The vagus nerve plays a key role in regulating central-autonomic interactions. Its stimulation influences widespread cortical and subcortical brain regions involved in cognition (Cakmak, 2019). Early animal studies demonstrated that vagus nerve stimulation (VNS) modulates cortical electrical activity and can disrupt pathological rhythmic discharges (Bailey and Bremer, 1938). In humans, systematic research on VNS began in the 1960s (Baldwin et al., 1966) and since the 1990s, its clinical application has been established for epilepsy and depression (Shafique and Dalsing, 2006). Therapeutic invasive VNS is now recommended in clinical guidelines for these conditions (Bundesärztekammer (Bäk), Kassenärztliche Bundesvereinigung (Kbv) and Arbeitsgemeinschaft Der Wissenschaftlichen Medizinischen Fachgesellschaften (Awmf), 2022; Ohemeng and Parham, 2020; Holtkamp et al., 2023; van Schooten et al., 2024).

Beyond seizure and mood control, invasive VNS has been associated with improvements in attention, concentration, and memory functions in patients with epilepsy (Englot et al., 2017; Orosz et al., 2014; Clark et al., 1999). Evidence suggests a time- and dose-dependent enhancement of verbal memory under chronic stimulation (Vonck et al., 2014). These cognitive effects are thought to arise from stimulation-induced activation of brainstem nuclei such as the locus coeruleus and the nucleus tractus solitarius, modulating noradrenergic and cholinergic signaling pathways that are crucial for learning and attention (Olsen et al., 2023).

However, invasive VNS requires surgical implantation and surgical and anesthetic risks as well as device-related complications (e.g., scarring, infection, bleeding) limit its broader use (Revesz et al., 2016).

In contrast, transcutaneous auricular vagus nerve stimulation (taVNS) represents a non-invasive alternative that targets the auricular branch of the vagus nerve through cutaneous stimulation at the external ear. It avoids surgical risks and can be applied repeatedly under standardized, well-tolerated conditions. TaVNS has gained growing interest as a potential therapeutic option in neuropsychiatry.

In healthy individuals, improvements in cognitive functions through taVNS are well documented and have been summarized in a systematic review by Ridgewell et al. (2021). However, evidence regarding cognitive effects of taVNS in individuals with neuropsychiatric disorders remains limited and heterogeneous, and no systematic review to date has specifically examined whether taVNS improves cognitive performance in clinical populations with neuropsychiatric disorders. Given that cognitive impairment is a core and clinically relevant feature across conditions such as epilepsy, major depressive disorder, Parkinson’s disease, and mild cognitive impairment (MCI), a focused synthesis of the available clinical evidence is warranted.

Cognitive deficits in neuropsychiatric disorders are currently addressed using pharmacological, behavioral, and neuromodulatory strategies, but their efficacy remains modest, domain-specific, and largely restricted to individual diseases rather than transdiagnostic application (Livingston et al., 2020). Pharmacological approaches, including acetylcholinesterase inhibitors, memantine, and monoaminergic agents, provide at best small to moderate cognitive benefits and are frequently limited by side effects, lack of durability, and poor generalizability across disorders and non-pharmacological interventions such as cognitive training and lifestyle-based approaches can improve selected cognitive domains but show variable transfer to everyday functioning and require sustained engagement (Venegas-Sanabria et al., 2024; Tahami Monfared et al., 2023). A recent umbrella reviews of other non-invasive brain stimulation techniques, repetitive transcranial magnetic stimulation and transcranial direct current stimulation, indicate an improvement of selected cognitive domains in specific conditions, but evidence is heterogeneous and disorder-restricted, which limits conclusions on broad, scalable cognitive efficacy (Wu et al., 2025). Consequently, no current intervention provides a well-tolerated, broadly effective, and scalable therapy for cognitive deficits across neuropsychiatric disorders, motivating evaluation of alternative neuromodulatory strategies such as taVNS. The present systematic review therefore aims to synthesize and critically evaluate current evidence on the effects of taVNS on global cognition and specific cognitive domains in individuals with neuropsychiatric disorders.

## 2 Methods

### 2.1 Study design

This systematic review followed the methodological standards of the Preferred Reporting Items for Systematic Reviews and Meta-Analyses (PRISMA) and the Synthesis Without Meta-analysis (SWiM) guidelines (Page et al., 2021; Campbell et al., 2020). The protocol was prospectively registered in the International Prospective Register of Systematic Reviews (PROSPERO CRD420251091407).

### 2.2 Population, intervention, comparator, and outcomes

Studies were eligible if they included adult participants with neuropsychiatric disorders. Studies that examined healthy individuals or participants without such disorders were excluded. The intervention of interest was taVNS, irrespective of specific stimulation site or parameters. Studies using transcutaneous cervical vagus nerve stimulation were excluded. Studies with a sham-taVNS control condition were included, as well as non-controlled studies assessing cognitive functions before and after active taVNS treatment. The primary outcome was cognition operationalized by global cognitive functioning and domain-specific cognitive functions.

### 2.3 Systematic review protocol

The review methodology was defined prior to data collection and adhered to PRISMA and SWiM recommendations. To ensure methodological quality and transparency, the review was evaluated using the AMSTAR-2 instrument (Shea et al., 2017). Of the 16 AMSTAR-2 items, 13 were applicable to this review, as no quantitative meta-analysis was conducted. Responses were rated as *yes*, *partly,* or *no*, and summarized in a traffic-light plot.

### 2.4 Search strategy

The systematic search was conducted on September 1, 2025. The search covered the period from database inception to September 1, 2025. Only articles published in English or German were eligible for inclusion. The following search string was applied:

(“cogniti*” OR “attenti*” OR “memor*” OR “language” OR “verbal” OR “visuo-spatial” OR “executi*” OR “dementia”) AND (“transcutaneous vagal nerve stimulation” OR “transcutaneous auricular vagal nerve stimulation” OR “transcutaneous vagus nerve stimulation” OR “transcutaneous auricular vagus nerve stimulation” OR “tVNS” OR “taVNS” OR “tcVNS” OR “t-VNS” OR “ta-VNS” OR “tc-VNS”) AND (“human” OR “humans”).

### 2.5 Data sources

Electronic searches were conducted in MEDLINE/PubMed, EMBASE, and the Cochrane Central Register of Controlled Trials (CENTRAL). CENTRAL also includes studies indexed in *clinicaltrials.gov*. These databases were selected according to Cochrane Collaboration recommendations for evidence synthesis in medical research (Higgins et al., 2016). In addition, a manual search was carried out following Cochrane guidance (Lefebvre et al., 2020). References from all included papers were screened to identify further relevant studies not captured in the database search. Reference lists of newly identified publications were screened recursively.

### 2.6 Study selection and data extraction

**Figure 1:**
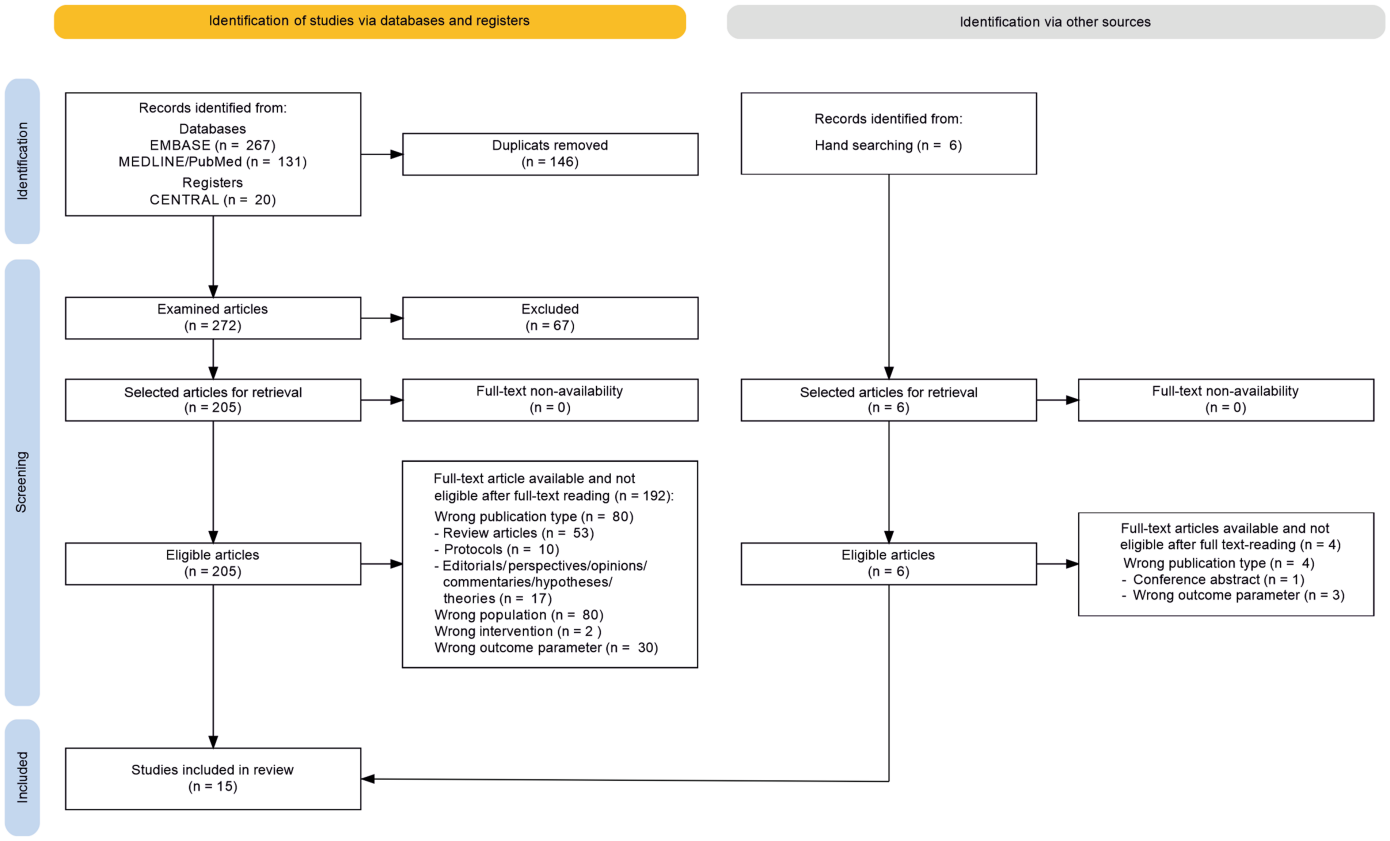
PRISMA flow diagram of study selection.

Study selection and data extraction were performed using the commercial software rayyan.ai (Ouzzani et al., 2016). The systematic search, study selection, and risk of bias assessments were independently conducted by two reviewers (H. D. and F. H.). Discrepancies regarding study eligibility or extracted data were resolved by consensus discussion between the two reviewers. The selection process was visualized in a PRISMA flow diagram (see Figure 1), comprising the stages of identification, screening, eligibility assessment, and inclusion (Ziegler et al., 2011). Database and manual search results were tracked separately.

### 2.7 Data analysis

For each included study, PICO parameters (Population, Intervention, Comparator, and Outcome) were extracted. Additional extracted variables included stimulation parameters (site, frequency, pulse width, intensity, daily stimulation time, total duration) in line with the international consensus recommendations for reporting taVNS research (Farmer et al., 2020).

Cognitive outcome variables were categorized according to the cognitive domains defined by the Movement Disorder Society (Litvan et al., 2012). These domains comprise attention and working memory, executive function, language, memory, and visuospatial function. In addition, the domain of social cognition was considered (Simpson, 2014). For each domain, it was recorded whether taVNS led to significant improvement.

In line with SWiM recommendations for systematic reviews, because of the heterogeneity of study designs and stimulation protocols, we summarized quantitative data using mode and range statistics rather than mean-based estimates. For measures of global cognition, Cohen’s *d* was calculated as effect size (Morris, 2008). Risk of bias was evaluated using the Cochrane Risk-of-Bias tool for randomized trials, which evaluates potential bias across five domains: randomization process, deviations from intended interventions, missing data, measurement of the outcome, and selection of the reported result (Savovic et al., 2014). For non-randomized studies, the ROBINS-I tool was applied, which assesses bias due to confounding, participant selection, intervention classification, deviations from intended interventions, missing data, outcome measurement, and selective reporting (Sterne et al., 2016). Results were presented in a traffic-light plot (Nejadghaderi et al., 2024). Financial or non-financial conflicts of interest of study authors were also extracted.

## 3 Results

The results of the database and registry searches are presented below.

### 3.1 Structured overview of relevant sources

Figure 1 illustrates the flow diagram of the literature search. There were no deviations from the protocol described in the Methods section. The systematic database and registry searches yielded the following results.

#### 3.1.1 Identification

A total of 418 records were identified. After removal of 146 duplicates, 272 unique records remained for screening.

#### 3.1.2 Screening

During the preliminary screening stage, 67 publications were excluded for the following reasons:

**Conference abstracts:** 58 records were excluded because they were conference proceedings and did not contain full peer-reviewed data (Albayrak et al., 2023; Aljuhani et al., 2023a; Austelle et al., 2021; Berlow, 2017; Bolender et al., 2024; Boon, 2015; Capone et al., 2022a; Capone et al., 2023; Capone et al., 2022b; Collier et al., 2022; Costa et al., 2022; Cribb et al., 2023; Davis and Lerman, 2016; De Gurtubay et al., 2022; De Smet et al., 2022; De Smet and Vanderhasselt, 2022; Dolphin et al., 2023a; Ellrich, 2012; Finisguerra et al., 2022; Genheimer et al., 2017b; Giraudier et al., 2023; Hinson et al., 2022; Jandackova et al., 2023a; Jandackova et al., 2023b; Jandackova et al., 2023c; Jandackova et al., 2019; Jandackova et al., 2020; Kainth et al., 2023; Keatch et al., 2023b; Konjusha et al., 2023b; Krishnamoorthy, 2023; Lerman et al., 2016; McGhee et al., 2023; McIntire et al., 2020; McIntire et al., 2021a; McKinley et al., 2019; McLeod et al., 2019; Moscote-Salazar and Zabaleta-Churio, 2016; Motolese et al., 2021; Nemechek and Nemechek, 2017; Pandža and Kuchinsky, 2021; Perez et al., 2024; Riegel et al., 2023; Sigrist et al., 2022; Silva-Jones et al., 2024; Slawson et al., 2022; Stefan et al., 2012a; Taylor et al., 2023; Tukaiev et al., 2022; Tyler, 2020; Vanderhasselt, 2022; Vasendova et al., 2019; Ventura-Bort et al., 2023; Verkuil et al., 2017a; Verkuil et al., 2017b; Warren et al., 2020; Zheng et al., 2023; Zhu et al., 2021).

**Corrigenda:** 2 publications were excluded because they were published as corrections (Hansen, 2019; McIntire et al., 2021b).

**Preprints:** 5 publications were excluded because they were preprints not yet peer-reviewed (Bahadori et al., 2024; Lloyd et al., 2023a; Lucchi et al., 2023; Tan et al., 2024a; Zhu et al., 2023).

**Foreign language:** 2 records written in Chinese were excluded due to language incompatibility (Ma et al., 2023; Zhang S. et al., 2022).

#### 3.1.3 Eligibility assessment of full texts

Full texts of 205 publications were retrieved and assessed for eligibility. All were accessible. After evaluation, 190 full texts were excluded for the reasons outlined below.

##### Incorrect publication type

**Review articles:** 53 publications were excluded because they were review papers rather than original research (Adair et al., 2020; Ahmed et al., 2022; Baptista et al., 2020; Boon et al., 2018; Bottari et al., 2024b; Bouwens van der Vlis et al., 2019; Broncel et al., 2020; Chen S. et al., 2023; Colzato and Beste, 2020; Colzato et al., 2022; de Carvalho et al., 2022; De Giorgio and Krahl, 2013; Décarie-Spain et al., 2024; Dedoncker et al., 2021; Doddamani et al., 2020; Dolphin et al., 2022; Farmer et al., 2020; Fawzy et al., 2023; Fernández-Hernando et al., 2023; Herrero Babiloni et al., 2023; Hirsch et al., 2016; Holland and Manning, 2022; Kaan and Lin, 2024; Krone et al., 2023; Li et al., 2023; Linnhoff et al., 2022; Liu et al., 2020; Ludwig et al., 2021; Mahmoud et al., 2023; Möbius and Welkoborsky, 2022; Mróz et al., 2022; Münchau et al., 2021; Naparstek et al., 2023; Nicholson et al., 2017; Ostergaard, 2023; Patel et al., 2022; Ridgewell et al., 2021; Riva et al., 2021; Ruhnau and Zaehle, 2021; Shen et al., 2022; Shou et al., 2021; Soltani et al., 2023; Soltani and Stavrakis, 2023; Trifilio et al., 2023; von Wrede and Surges, 2021; Wan et al., 2024; Wang et al., 2022a; Wang et al., 2021; Wang et al., 2023b; Yakunina and Nam, 2021; Yang J. et al., 2023; Zaehle and Krauel, 2021; Zhu et al., 2022b).

**Protocols and trial registrations:** 10 publications were excluded because they represented study protocols or trial registrations (Bottari et al., 2024b; Dolphin et al., 2023b; University of California San Francisco, 2019; Kamboj et al., 2023; Li et al., 2022; Liu et al., 2024; Gierthmuehlen et al., 2023; Sun et al., 2024; Trevizol et al., 2015; Zhang Z. Q. et al., 2022).

**Editorials, perspectives, opinions, comments, hypotheses, or theoretical papers:** 17 publications were excluded for this reason (Briand et al., 2020; Carreno and Frazer, 2016; Colzato et al., 2023a; Colzato et al., 2023b; Cota and Moraes, 2022; De Martino et al., 2021; Forte et al., 2022; Hansen, 2018; Hilz and Bolz, 2022; Janitzky, 2020; Jin and Kong, 2017; Konjusha et al., 2022a; Slavin, 2024; Weymar and Zaehle, 2021; Xiong et al., 2009; Zaehle et al., 2021; Zou et al., 2022).

**Incorrect study population:** 80 publications were excluded because they investigated healthy participants or other populations without neuropsychiatric disorders (Alicart et al., 2021; Aljuhani et al., 2023b; Azabou et al., 2021; Beste et al., 2016; Borges et al., 2020; Borges et al., 2021; Burger et al., 2020; Burger et al., 2019a; Burger et al., 2019b; Burger et al., 2017; Burger et al., 2016; Capone et al., 2021; Chen et al., 2024; Chen et al., 2023a; Chen et al., 2023b; Colzato et al., 2018a; Colzato et al., 2017; Colzato et al., 2018b; De Smet et al., 2021; De Smet et al., 2023; Ferstl et al., 2022; Finisguerra et al., 2019; Fischer et al., 2018; Gadeyne et al., 2022; Genheimer et al., 2017a; Gurtubay et al., 2023; Jacobs et al., 2015; Janner et al., 2018; Johnson and Steenbergen, 2022; Kaan et al., 2021; Keatch et al., 2023a; Konjusha et al., 2022b; Konjusha et al., 2023a; Kühnel et al., 2020; Le Roy et al., 2023; Lee et al., 2024; Liao et al., 2023; Llanos et al., 2020; Lloyd et al., 2023b; Lucchi et al., 2024; Maraver et al., 2020; McHaney et al., 2023; McIntire et al., 2021c; Obst et al., 2022; Obst et al., 2020; Phillips et al., 2021; Pihlaja et al., 2020; Rufener et al., 2018; Sellaro et al., 2018; Sellaro et al., 2015; Sharon et al., 2021; Sommer et al., 2023; Steenbergen et al., 2020; Steenbergen et al., 2021; Steenbergen et al., 2015; Sun et al., 2021; Szeska et al., 2020; Szeska et al., 2021; Tan et al., 2024b; Teckentrup et al., 2021; Thakkar et al., 2020; Thakkar et al., 2023; Tian et al., 2023; Tona et al., 2020; Van Leusden et al., 2015; van Midden et al., 2024; Ventura-Bort et al., 2018; Ventura-Bort et al., 2021; Verkuil and Burger, 2019; Villani et al., 2022; Warren et al., 2019a; Wienke et al., 2023; Wolf et al., 2021; Yıldız et al., 2023; Zhang L. et al., 2023; Zhang S. et al., 2023; Zhao et al., 2023; Zhao et al., 2022; Zhou et al., 2022; Zhu et al., 2022a).

**Incorrect intervention:** 2 studies were excluded because they used transcutaneous cervical instead of auricular vagus nerve stimulation (Zhang et al., 2023b; Choudhary et al., 2023).

**Incorrect outcome variables:** 30 publications were excluded because they did not include neuropsychological or cognitive outcome measures (Bauer et al., 2016; Black et al., 2023; Bottari et al., 2024a; Bremner et al., 2020; Gazi et al., 2022; Gazi et al., 2023; Gurel et al., 2020; Hakon et al., 2020; Koenig and Vöckel, 2024; Kreuzer et al., 2014; Liu et al., 2016; Long et al., 2020; Manning et al., 2019; Murphy et al., 2023; Osińska et al., 2022; Rao et al., 2023; Sabé et al., 2023; Sfera et al., 2023; Shiraishi et al., 2024; Stefan, 2014; Sun et al., 2023; Sun et al., 2022; Tseng et al., 2022; von Wrede et al., 2019; Wang et al., 2024; Wang et al., 2023a; Yi et al., 2022; Yu et al., 2017; Yuan et al., 2019; Zuo et al., 2023).

##### Manual search

An additional manual search identified six further records. Among these, one was a conference abstract (Nemechek et al., 2017) and three lacked cognitive outcome variables (Zhang et al., 2023a; Marano et al., 2024; Torrecillos et al., 2022) and were therefore excluded. The remaining two studies (Marano et al., 2022; Corrêa et al., 2022) were included in the final analysis.

#### 3.1.4 Included studies

A total of 15 publications met all inclusion criteria and were included in this systematic review: (Corrêa et al., 2022; Evensen et al., 2022; Lench et al., 2023; Marano et al., 2022; Mertens et al., 2022; Oehrn et al., 2022; Pan et al., 2024; Stefan et al., 2012b; Trevizol et al., 2016; Uehara et al., 2022; von Wrede et al., 2021; Wang et al., 2022b; Weber et al., 2021; Yang H. et al., 2023; Zheng et al., 2024).

### 3.2 Study results and critical appraisal

The PICO parameters (population, intervention, comparator, outcomes) of the 15 included studies are summarized in Table 1. Table 2 presents similarities and differences in stimulation parameters and treatment across studies. A synthesis of the cognitive outcomes associated with taVNS is summarized in Table 3.

**Table 1:** Population, intervention, comparator, and clinical outcomes.

Author, year, country	Study design/type	NHMRC level of evidence	Diagnosis of study population	Sample size at inclusion active / control	Intervention	Comparator	Cognitive outcome measures
Pan et al. (2024), China	RCT (double-blind, sham-controlled)	II	Epilepsy	19/9	taVNS	Sham taVNS	Reaction time in a delayed visual matching task
Uehara et al. (2022), Brazil	RCT (double-blind, sham-controlled)	II	COVID-19	10/11	taVNS	Sham taVNS	Clinical Global Impression: Improvement Scale for memory and attention (14 days post-intervention, including telephone assessment)
Corrêa et al. (2022), Brazil	RCT (single-blind for participants, sham-controlled)	II	COVID-19	26/26 post-intervention	taVNS	Sham taVNS	Clinical Global Impression Scale for symptom burden of memory and attention (every 30 days up to 180 days post-intervention, including telephone assessment)
Marano et al. (2022), Italy	RCT (double-blind, sham-controlled, cross-over design)	II	Parkinson’s disease	12/12	taVNS	Sham taVNS	Flanker test
Wang et al. (2022b), China	RCT (double-blind, sham-controlled)	II	Mild cognitive impairment	25/27	taVNS	Sham taVNS	1. MoCA, 2. Auditory Verbal Learning Test, 3. Shape Trails Test, 4. Animal fluency test, 5. Boston Naming Test
Evensen et al. (2022), Denmark	Non-RCT	IV	Major depression	20 / non-controlled	taVNS	Non-controlled	Cognitive processing speed (a quick cognitive test of cognitive speed)
Lench et al. (2023), USA	RCT (double-blind, sham-controlled)	II	Parkinson’s disease	15/15	taVNS	Sham taVNS	1. Digit Span test (forward and backward), 2. Delis-Kaplan Executive Function System (phonemic, semantic, and category switching)
Mertens et al. (2022), Germany	RCT (double-blind, sham-controlled, cross-over design)	II	Epilepsy	15/15	taVNS	Sham taVNS	Experimental paradigm assessing verbal working memory
Weber et al. (2021), Germany	RCT (double-blind, sham-controlled, cross-over design)	III-1	Epilepsy	8/8	taVNS	Sham taVNS	Experimental paradigm assessing reward learning
Oehrn et al. (2022), Germany	RCT (double-blind, sham-controlled, cross-over design)	II	Epilepsy	19/19	taVNS	Sham taVNS	Experimental paradigm assessing social cognition in the Prisoner’s Dilemma task
Stefan et al. (2012b), Germany	Non-RCT	IV	Epilepsy	10 / non-controlled	taVNS	Non-controlled	Computer-based assessment of attention, working memory, cognitive processing speed, and verbal and spatial memory
Trevizol et al. (2016), Brazil	Non-RCT	IV	Major depression	12 / non-controlled	taVNS	Non-controlled	MoCA
Von Wrede et al. (2021), Germany	Non-RCT	IV	Epilepsy	14 / non-controlled	taVNS	Non-controlled	1. EpiTrack (3rd edition) for attention and executive functions, 2. Rey Auditory Verbal Learning Test
Yang H. et al. (2023), China	RCT (double-blind, sham-controlled)	II	Epilepsy	76/36	taVNS	Sham taVNS	MoCA
Zheng et al. (2024), USA	Non-RCT	IV	Long COVID	24 / non-controlled	taVNS	Non-controlled	1. Flanker Test, 2. Dimensional Change Card Sorting Test, 3. Picture Sequencing Memory Test, 4. List Sorting Working Memory Test, 5. Pattern Comparison Processing Speed Test

Hz, Hertz; mA, milliampere; ms, millisecond; MoCA, Montreal Cognitive Assessment; RCT, Randomized controlled trial; taVNS, transcutaneous auricular vagus nerve stimulation.

**Table 2:** Stimulation settings and duration.

Study	Duration of daily stimulation session (minutes)	Total taVNS treatment duration	Age (mean (SD) or range, years) active / control	Stimulation site	Pulse width (ms)	Intensity (SD, range, or protocol-defined value)	Frequency (Hz)
Pan et al. (2024)	90–150	140 consecutive days	37.0 (11.6) / 42.0 (9.7)	Left cymba conchae	0.25	30–50 Volt	25
Uehara et al. (2022)	180	7 consecutive days	53 (10.8) / 44 (22.7)	Left tragus	Not reported	0.5–20 mA	30
Corrêa et al. (2022)	180	7 consecutive days	53 (17) / 57 (16)	Left tragus	1	Not reported	25
Marano et al. (2022)	30	7 consecutive days	75.5 (7.1)	Left tragus	0.3	Not reported	20
Wang et al. (2022b)	60	120 non-consecutive days over 148 days	66.9 (3.7) / 67.0 (4.4)	Left cymba conchae and scapha	Not reported	0.6–1.0 mA	20–100
Evensen et al. (2022)	216 (54)	28 consecutive days	49.4 (11.2) / non-controlled	Left cymba conchae	Not reported	1.1 mA (0.9)	25
Lench et al. (2023)	60	10 non-consecutive days within 14 days	65.4 (7.6) / 68.4 (7.6)	Left tragus	0.5	2.0 mA (0.5)	25
Mertens et al. (2022)	6	1 day (assessment of immediate effects)	39.5 (12.6)	Left cymba conchae	0.25	2.4 mA (1.2)	25
Weber et al. (2021)	180	2 days of stimulation separated by at least 2 weeks	43.9 (10.9)	Left cymba conchae	Not reported	1.3 mA (0.6)	25
Oehrn et al. (2022)	120–240	1 day (assessment of immediate effects)	45 (12)	Left cymba conchae	Not reported	1.2 mA (0.5)	25
Stefan et al. (2012b)	180	270 consecutive days	37.7 (18–55)	Auricular branch of the left vagus nerve, exact location not reported	0.3	25 Volt	Not reported
Trevizol et al. (2016)	30	10 non-consecutive days within 14 days	45.9 (9)	Bilateral auricular branch of the vagus nerve, exact location not reported	0.25	12 mA	120
Von Wrede et al. (2021)	60	1 day (assessment of immediate effects)	41.1 (22.2)	Left cymba conchae	Not reported	2.0 (1.0)	25
Yang H. et al. (2023)	120	140 consecutive days	33.3 (11.3)	Left cymba conchae	0.25	Not reported	25
Zheng et al. (2024)	60	10 consecutive days	45.8 (11.7)	Left tragus	0.25	13.6 mA	25

Hz, Hertz; mA, milliampere; ms, millisecond; taVNS, transcutaneous auricular vagus nerve stimulation.

**Table 3:** Efficacy of transcutaneous auricular vagus nerve stimulation on global cognition or individual cognitive domains and risk of bias.

Study	Global cognition and/or individual cognitive domains	Did transcutaneous auricular vagus nerve stimulation significantly improve this outcome variable?	Risk of bias (study-level assessment)
Pan et al. (2024)	Attention, Memory	Yes	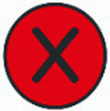
Uehara et al. (2022)	1. Attention 2. Memory	1. No 2. No	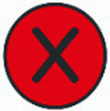
Corrêa et al. (2022)	Attention, Memory	No	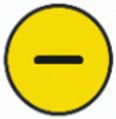
Marano et al. (2022)	Attention, Executive function	Yes	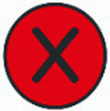
Wang et al. (2022b)	1. Global cognition 2. Memory 3. Attention (Cognitive processing speed) 4. Executive function (Cognitive flexibility) 5. Language	1. Yes 2. Yes 3. No 4. Yes 5. Yes	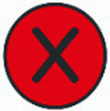
Evensen et al. (2022)	Attention	Yes	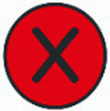
Lench et al. (2023)	1. Working memory 2. Executive function (Phonemic fluency) 3. Memory 4. Language (Category fluency)	1. No 2. No (significant worsening) 3. No 4. No (significant worsening)	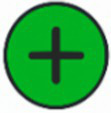
Mertens et al. (2022)	1. Attention 2. Memory	1. No 2. Yes	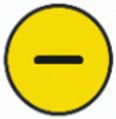
Weber et al. (2021)	1. Attention: Non-decision time 2. Executive function: Reward-based learning (accuracy)	1. Yes 2. Yes	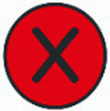
Oehrn et al. (2022)	Social cognition	Yes (behavioral effect)	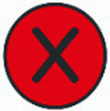
Stefan et al. (2012b)	Attention, Working memory, Memory	No	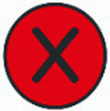
Trevizol et al. (2016)	Global cognition	No	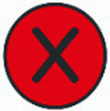
Von Wrede et al. (2021)	1. Attention and Executive function 2. Verbal memory	1. No 2. No	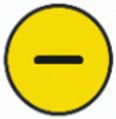
Yang H. et al. (2023)	Global cognition	No	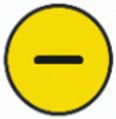
Zheng et al. (2024)	1. Attention 2. Executive function 3. Episodic memory 4. Working memory 5. Attention (Cognitive processing speed)	1. Yes 2. No (trend only) 3. Yes 4. Yes 5. Yes	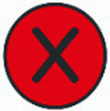

Green circles indicate low risk of bias, yellow circles indicate moderate risk of bias, and red circles indicate high risk of bias. Cognitive outcomes and risk of bias assessments are reported independently; risk of bias ratings refer to overall study quality and not to individual outcome measures. taVNS, transcutaneous auricular vagus nerve stimulation.

Supplementary Table 1 summarizes adverse events across the included studies. Nine of the fifteen studies (60%) explicitly reported (serious) adverse events monitoring or occurrence, while six (40%) did not provide safety information. Reported adverse events were mild and transient, and no serious adverse events or adverse events with sequelae occurred in any study.

### 3.3 Summary of findings across included studies

Using mode statistics across Tables 1–3, commonalities among the 15 included studies can be summarized as follows:

- **Population:** The most frequent patient population was epilepsy.
- **Design/comparator:** The most frequent design was a randomized controlled trial comparing active taVNS with sham taVNS.
- **Primary outcome measure:** The Montreal Cognitive Assessment (MoCA) was the most frequently used outcome measure, though only in three studies in total. When individual outcome measures were grouped into cognitive domains, attention was assessed most often, i.e., more frequently than global cognition.
- **Stimulation site:** The cymba conchae was the most frequently stimulated target.
- **Timing and dose:** Immediate (acute) effects were most commonly investigated, most often with 60 min/day of stimulation.
- **Parameters:** The most frequent settings were 250 μs pulse width and 25 Hz frequency. Stimulation intensity was most commonly not reported; on re-inspection of all included papers, authors most frequently stated that intensity was titrated between perception and pain thresholds.
- **Geography:** Studies were most commonly conducted in Germany.

Between-study differences are quantifiable by the reported ranges: 1–270 days of stimulation, 6–240 min/day, and wide parameter spans (pulse width 0.25–1.00 ms; frequency 20–120 Hz; intensity 0.6–13.6 mA or 30–50 V). Target regions on the ear also varied, with stimulation most often at the left ear, typically the tragus or cymba conchae.

Across the 15 included studies, 8 reported statistically significant improvements in global cognition or at least one specific cognitive domain following taVNS, while 7 reported no significant cognitive benefit. Improvements were most frequently observed in attention and working memory (reported in 5 studies), followed by memory (4 studies), executive functions (3 studies), language (1 study), social cognition (1 study), and global cognition (1 study). For each cognitive domain, at least one study also reported no effect, underscoring the heterogeneity of findings across populations, outcome measures, and study designs.

For global cognition, three studies used the MoCA (Trevizol et al., 2016; Wang et al., 2022b; Yang H. et al., 2023). Cohen’s d was small in Trevizol et al. (2016), moderate in Wang et al. (2022b), and not computable for Yang H. et al. (2023), because results were presented only graphically without clear standard deviations.

Risk of bias was high in the majority of included studies (see Table 3). Only one study showed low risk of bias (Lench et al., 2023), and four studies were rated as having some concerns (Corrêa et al., 2022; Mertens et al., 2022; von Wrede et al., 2021; Yang H. et al., 2023).

### 3.4 Positioning the included literature within the taVNS field

The stimulation parameters observed here align with those summarized by Ridgewell et al. (2021) in a review of 19 studies in healthy adults, where 0.25 ms pulse width and 25 Hz frequency were also most frequent. In neuropsychiatric populations, the present review found attention to be the most commonly studied domain; by contrast, in healthy adults, executive functions were investigated most frequently, followed by attention (Ridgewell et al., 2021). Ridgewell et al. further noted a wide dispersion of outcome measures across studies in healthy adults, with no overlap of identical outcomes between studies. Their meta-analysis suggested that taVNS improves executive functions in healthy adults, while effects on attention and memory were not observed (Ridgewell et al., 2021). A subgroup analysis across nine publications indicated that left tragus stimulation yielded greater efficacy than left cymba conchae (g = 0.48; Ridgewell et al., 2021). They also reported a small but significant effect on global cognitive performance (g ≈ 0.21). The influence of baseline global and executive performance was not reported in that review.

### 3.5 Quality control of the present review using AMSTAR-2

Table 4 summarizes the methodological quality assessment of the present review using the AMSTAR-2 instrument. The 16 AMSTAR-2 items served as a structured checklist to systematically verify the methodological rigor and transparency of the review process. Each item was linked to the corresponding section in the manuscript. As the review did not include a quantitative meta-analysis, items 11, 12, and 15 were not applicable. Most domains (e.g., PICO definition, search strategy, study selection and data extraction, and risk-of-bias assessment) were rated positively, indicating good methodological transparency. The results are visualized in a traffic-light plot.

**Table 4:** Quality control of the present review using AMSTAR-2 including traffic light plot.

AMSTAR-2 item	Corresponding section in review	Quality rating (Traffic light)
D1	see *Results* 3.2 (critical appraisal) and Tables	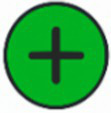
D2	see *Methods* 2.1–2.7 and *Results* 3.1 (flow/structured sources).	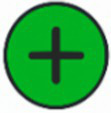
D3	see *Methods* 2.2–2.4 (participants/interventions/comparators) and inclusion criteria	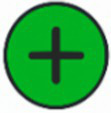
D4	see *Methods* 2.5–2.7.	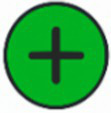
D5	see *Methods* 2.8	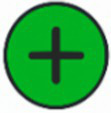
D6	see *Methods* 2.8	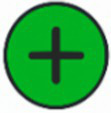
D7	see *Results* 3.1.3 (full-text eligibility reasons)	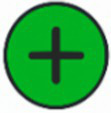
D8	see *Results* 3.3 and Tables	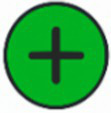
D9	see *Methods* 2.9, *Results* 3.3 (risk-of-bias summary), and Table 3	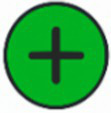
D10	see *Discussion*, 4.3	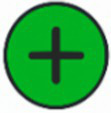
D11	not applicable	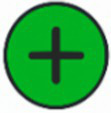
D12	not applicable	
D13	see *Methods* 2.9 and *Results* 3.3 (risk-of-bias summary).	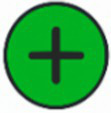
D14	see *Discussion* (limitations: outcome heterogeneity).	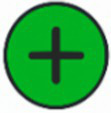
D15	not applicable	
D16	see *5 Conflict of Interest*	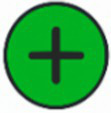

1. Did the research questions and inclusion criteria for the review include the components of PICO (population, intervention, comparator, outcomes)? 2. Did the report of the review contain an explicit statement that the review methods were established prior to the conduct of the review and did the report justify any significant deviations from the protocol? 3. Did the review authors explain their selection of the study designs for inclusion in the review? 4. Did the review authors use a comprehensive literature search strategy? 5. Did the review authors perform study selection in duplicate? 6. Did the review authors perform data extraction in duplicate? 7. Did the review authors provide a list of excluded studies and justify the exclusions? 8. Did the review authors describe the included studies in adequate detail? 9. Did the review authors use a satisfactory technique for assessing the risk of bias in individual studies that were included in the review? 10. Did the review authors report on the sources of funding for the studies included in the review? 11. If meta-analysis was performed, did the review authors use appropriate methods for statistical combination of results? 12. If meta-analysis was performed, did the review authors assess the potential impact of risk of bias in individual studies on the results of the meta-analysis or other evidence synthesis? 13. Did the review authors account for risk of bias in individual studies when interpreting/discussing the results of the review? 14. Did the review authors provide a satisfactory explanation for, and discussion of, any heterogeneity observed in the results of the review? 15. If they performed quantitative synthesis (meta-analysis), did the review authors carry out an adequate investigation of publication bias (small study bias) and discuss its likely impact on the results of the review? 16. Did the review authors report any potential sources of conflict of interest, including any funding they received for conducting the review?

## 4 Discussion

In this systematic review combining database and registry searches with predefined keywords and an additional manual search, 15 publications investigating the cognitive effects of taVNS in patients with neuropsychiatric disorders were identified. Across these studies, cognitive outcomes were heterogeneous. While 8 of 15 publications reported improvements in global cognition or specific cognitive domains following taVNS, several studies reported null or mixed effects, indicating that cognitive benefits are not uniform across populations, domains, or study designs. Regarding the primary research question, improvements were reported in at least one study for the following domains: (1) global cognition (Wang et al., 2022b), (2) attention and working memory (Marano et al., 2024; Zheng et al., 2024; Evensen et al., 2022; Weber et al., 2021; Pan et al., 2024), (3) memory (Wang et al., 2022b; Pan et al., 2024; Zheng et al., 2024; Mertens et al., 2022), (4) executive functions (Marano et al., 2024; Wang et al., 2022b; Weber et al., 2021), (5) language (Wang et al., 2022b), and (6) social cognition (Oehrn et al., 2022).

Placing these findings into a broader clinical context, a recent systematic review and meta-analysis primarily focusing on motor outcomes of taVNS in Parkinson’s disease also assessed cognitive effects as secondary outcomes (Shan et al., 2025). While beneficial effects were reported for several motor parameters, cognitive outcomes were heterogeneous and partly unfavorable, including impairments in verbal fluency. These findings suggest that cognitive effects of taVNS in Parkinson’s disease are not uniformly beneficial and may depend on the cognitive domain assessed, disease-specific factors, and stimulation parameters.

### 4.1 Heterogeneity of population, intervention, and comparator

Study populations differed: 7 studies included patients with epilepsy, 3 with COVID-19/long-COVID, 2 with Parkinson’s disease, 2 with major depression, and 1 with MCI. The intervention in all included studies was taVNS. Of the included studies, two-thirds were sham-controlled, and one-third were uncontrolled. The largest study was randomized, controlled, and double-blind, with 76 patients with epilepsy in the active arm and 36 in the control arm (Yang H. et al., 2023). All other studies treated fewer than 30 patients with taVNS.

### 4.2 Outcome heterogeneity

Interpretation is limited by heterogeneity of outcomes and scales. The most frequently assessed measure, the MoCA, was collected in only three studies; of these, only one study in MCI reported improvement with taVNS, whereas the other two showed no change. The study by Yang H. et al. (2023) with 76 active and 36 sham-treated epilepsy patients found no improvement in global cognition. Comparing MCI and epilepsy cohorts, baseline differences were evident in MoCA scores and age (Wang et al., 2022b, Yang H. et al., 2023). In MCI, the pre-treatment mean MoCA was approximately 19.5 points (graphically reported only), versus 20.6 in epilepsy; age differed more substantially (66.9 years in MCI versus 33.3 in epilepsy). Such clinical and demographic differences may confound and contribute to outcome heterogeneity.

A quantitative heterogeneity analysis is not informative given the small number of studies. Because cognitive domains were tested with different instruments, domain-specific comparisons are also restricted. A potential approach would convert study outcomes to norm-referenced metrics per test and then compare across cohorts; this could be pursued in future work, acknowledging possible trade-offs in interpretability and clinical utility.

### 4.3 Financial conflicts of interest and research funding

Author honoraria indicating financial conflicts of interest were reported in 2 of 15 included publications (≈13%), which is not negligible. Nevertheless, with >85% investigator-initiated research, the field is not dominated by financial interests. To advance the field, public funding (e.g., from the German Federal Ministry of Education and Research, the German Research Foundation, the European Union’s Horizon Europe program, or the US National Institutes of Health) would be desirable but has not yet been provided for taVNS. Industry partnerships could also be beneficial but require financially strong partners and independent scientific steering groups. Major device manufacturers (Boston Scientific, Medtronic, Abbott) have not entered the taVNS market to date; in Germany and Europe, the market is dominated by tVNS Technologies, whose taVNS devices are CE-certified. With less than 10 million € in annual revenue and less than 20 employees (Duphorn, 2024), tVNS Technologies cannot fund large multicenter RCTs comparable to those supported by global leaders in deep brain stimulation (Deuschl et al., 2006; Schuepbach et al., 2013).

### 4.4 Possible mechanisms of action

Potential mechanisms underlying the observed cognitive effects are discussed below. Building on the theoretical background, we distinguish direct neurophysiological mechanisms closely linked to neuroanatomy/physiology from general mechanisms with system-level or medication-related origins.

#### 4.4.1 Direct neurophysiological effects of taVNS

**Stimulation of the vagus-solitary complex:** Via afferent fibers of the auricular branch of the vagus nerve, taVNS activates the vagus-solitary complex in the brainstem (Komisaruk and Frangos, 2022), a hub integrating visceral and gustatory input and implicated in memory for experiences that elevate central arousal (Kerfoot et al., 2008).

**Activation of adjacent cranial nerve nuclei:** taVNS may co-activate nuclei neighboring the vagus-solitary complex, e.g., the nucleus tractus solitarii (Engineer et al., 2019), which contributes to memory formation (Garcia-Medina and Miranda, 2013).

**Modulation of brain networks:** Through its projections, the vagus-solitary complex influences distributed regions: nucleus parabrachialis, substantia nigra, trigeminal nucleus, locus coeruleus, red nucleus, cerebellum, bed nucleus of the stria terminalis, amygdala, nucleus accumbens, insula, and pre/postcentral gyri (Komisaruk and Frangos, 2022). These regions subserve specific cognitive functions: attention (locus coeruleus; Sara, 2009); visuospatial functions (nucleus basalis of Meynert, insula; Gratwicke et al., 2015); executive functions (fronto-striatal networks modulated by substantia nigra; Gratwicke et al., 2015); and memory (medial temporal lobe, including amygdala; Gratwicke et al., 2015). Network-level modulation by taVNS may thus contribute to domain-specific cognitive effects.

**Modulation of multiple neurotransmitter systems:** Auditory evoked-potential work indicates modulation of GABA (gamma-band frequency/power), acetylcholine (sensory gating), serotonin (loudness dependence of auditory evoked potentials), and noradrenaline (P300b; Lewine et al., 2019). Additional biomarker studies corroborate these effects using motor-evoked potentials for cholinergic function (Horinouchi et al., 2024), salivary alpha-amylase or pupillary dilation for noradrenergic tone (Reimer et al., 2016; Warren et al., 2019b), TMS for GABAergic function (van Midden et al., 2023), and fMRI for serotonergic pathways (Borgmann et al., 2021). Analogous to “dirty drugs” with multi-target actions that can yield broader efficacy (Abbenante et al., 2008), multi-system modulation by taVNS may be advantageous—while remaining safe and well-tolerated (Ridgewell et al., 2021).

#### 4.4.2 Cognitive control mechanisms: sensory gaiting and attentional filtering

Cognition relies on filtering and routing of lower-level information to higher-order processes (Chrysikou et al., 2014). Sensory gating is one such filter, selecting salient sensory/auditory inputs and supporting cognitive control (Chrysikou et al., 2014). The present review found taVNS can improve cognitive deficits in Parkinson’s disease, major depression, epilepsy, and MCI, which are conditions with known sensory gating disturbances (Geyer, 2006). Although sensory gating in COVID-19 has not been systematically studied, taVNS has been shown to improve sensory gating (Lewine et al., 2019), which could contribute to observed cognitive benefits. Further studies should examine links between improved gating and cognitive outcomes in neuropsychiatric populations treated with taVNS.

#### 4.4.3 Indirect clinical effects via medication reduction

TaVNS is effective in several neuropsychiatric conditions. In epilepsy, it can reduce seizure frequency and thereby allow lowering antiepileptic drugs (e.g., zonisamide, benzodiazepines) that frequently impair cognition (Katja Eva, 2020). In migraine/cluster headache, taVNS can reduce attack frequency and enable tapering of prophylactics such as topiramate, which can adversely affect cognition (Brandt et al., 2015). Such medication reductions may improve cognitive side effects—an indirect yet clinically relevant “net effect” of taVNS.

### 4.5 Technical considerations on systems, sites, and parameters

Across studies, 250 μs pulse width and 25 Hz frequency were most common, with stimulation intensity set just below pain threshold. fMRI data indicate that taVNS with these parameters suffices to modulate brainstem activity and distant but functionally connected regions, e.g., dorsolateral prefrontal cortex (Dietrich et al., 2008). Several technical issues warrant further research. The optimal stimulation site remains unsettled, and the review revealed substantial heterogeneity in outcomes, parameters, and sites. Future studies should examine how site and settings relate to cognitive effects. taVNS is commonly applied to the left ear—historically inherited from cervical VNS, where right-sided stimulation risks cardiac arrhythmias via efferent vagal fibers (Nemeroff et al., 2006). For taVNS, only afferent fibers projecting to the brainstem are stimulated (Kreuzer et al., 2012), and arrhythmias are not expected with right-sided stimulation; indeed, Trevizol et al. (2016) reported no arrhythmias even with bilateral taVNS.

### 4.6 Comparison of effect sizes with other therapies

Among the three studies assessing taVNS effects on global cognition (Trevizol et al., 2016; Wang et al., 2022b; Yang H. et al., 2023), only the MCI cohort showed a significant, moderate effect (Wang et al., 2022b). By contrast, a meta-analysis of cognitive training in MCI reported large effects with three studies and 199 participants and low heterogeneity (Mamayson and Lacanaria, 2024).

### 4.7 Limitations

This review combined systematic database/registry searches with a manual search, including recursive reference screening and use of a personal reference database as described in Methods. A limitation arises from the nomenclature variability of taVNS. We searched multiple formulations (e.g., “transcutaneous vagus/vagal nerve stimulation,” with and without “auricular”), but numerous further variants are conceivable (e.g., “transauricular…,” “auricular non-invasive…”). To mitigate this, three strategies were applied: (1) acronym use (with/without hyphenation to “VNS”), (2) guideline-recommended terminology, and (3) a complementary manual search.

The AMSTAR-2 served as a practical framework for quality control across introduction, methods, results, and discussion. Moreover, the interpretation of the present findings is limited by the methodological quality of the available studies. The majority of included studies were characterized by small sample sizes, heterogeneous designs, and a high or unclear risk of bias. Only one study was rated as having low risk of bias, while most showed either some concerns or high risk across key domains. Such limitations substantially reduce statistical power, increase susceptibility to type I and type II errors, and limit the generalizability of reported effects. Consequently, the current evidence does not allow definitive conclusions regarding the clinical effectiveness of taVNS for cognitive deficits.

Further, insufficient studies were available to conduct a quantitative synthesis of findings by means of meta-analysis. Meta-regression on dose–response effects would be highly informative for the field, informing stimulation parameters and clinical dosing protocols.

### 4.8 Implications and recommendations

This review indicates that taVNS can improve global cognition and specific domains (attention, memory, executive functions, language, and social cognition) in disorders such as major depression, epilepsy, Parkinson’s disease, MCI, and COVID-19. The range of clinical efficacy appears broad and may reflect demographic/clinical differences (age, disease duration, ethnicity, baseline cognitive severity). To increase impact and clarity, future research directions can be prioritized and grouped into four thematic areas: (i) patient stratification and biomarkers, (ii) harmonization of outcomes and study design, (iii) mechanistic and translational research, and (iv) long-term efficacy, technical optimization, and multimodal interventions.

- **Patient stratification and biomarkers:** First, future studies should prioritize analyses of demographic, clinical, and imaging-based predictors of cognitive effects of taVNS, as established in our Cologne group for other neuromodulation modalities such as deep brain stimulation (Loehrer et al., 2024; Jost et al., 2021; Sauerbier et al., 2021; Irmen et al., 2020; Petry-Schmelzer et al., 2019). Imaging predictors may include connectomics, voxel-based morphometry, DTI-based microstructure, and volume-of-tissue-activated analyses. Baseline cognitive burden should be incorporated as an inclusion or stratification criterion (e.g., in Parkinson’s disease, require defined executive dysfunction measured by Stroop or Wisconsin Card Sorting Test).
- **Harmonization of outcomes and study design:** Second, interdisciplinary collaboration across disease areas should be strengthened to harmonize neuropsychological batteries and outcome measures, enabling an identification of convergent taVNS effects on shared cognitive domains and improving cross-study comparability. Stronger interdisciplinary collaboration across neurology, psychiatry, psychology, and cognitive neuroscience is required to establish such standardized frameworks and to identify convergent cognitive effects of taVNS across neuropsychiatric disorders.
- **Mechanistic and translational research:** Third, mechanistic studies should integrate molecular biomarkers to elucidate pathways of action, e.g., BDNF and spatial transcriptomics of splenic T-cells. Future work should also establish robust animal models of taVNS that closely mirror human stimulation sites and dosing and integrate molecular readouts with whole-brain imaging. Pairing transcriptomic, proteomic, and inflammatory markers with modalities such as fMRI or fiber photometry will help link peripheral neuromodulatory effects to circuit-level changes and, ultimately, to cognitive outcomes.
- **Long-term efficacy, technical optimization, and multimodal interventions:** Fourth, future trial should extend investigation to additional disorders characterized by deficits in attention, memory, and executive function including ADHD, Alzheimer’s disease, frontotemporal dementia, left-hemispheric stroke with apraxia.

For neurodegenerative or partially degenerative disorders, trials should extend observation windows to at least 1–2 years to capture medium- to long-term efficacy on cognition. Studies with durations of 5 years or longer are needed to evaluate sustained effectiveness, durability of response after parameter adjustments, and long-term safety, including adherence and device tolerability issues.

Technical advances are warranted to improve ear-conforming electrodes that deliver current more efficiently and reproducibly across auricular targets. Parameter optimization—particularly pulse width, frequency duty cycles, and daily dose—should be pursued alongside secure remote programming capabilities to enable supervised, telemedicine-based titration and monitoring.

Finally, taVNS should be evaluated within multimodal treatment strategies that pair neuromodulation with pharmacological and non-pharmacological interventions known to improve cognition, such as acetylcholinesterase inhibitors, structured cognitive training, or light therapy (Alves et al., 2013; Huang et al., 2024). Synergistic designs may enhance effect sizes and broaden clinical applicability across disease stages.

## 5 Conclusion

In conclusion, the available evidence suggests that taVNS is a safe and well-tolerated intervention with potential beneficial effects on cognition in neuropsychiatric disorders. However, given the heterogeneity of findings, small sample sizes, and generally high risk of bias, the current evidence base remains insufficient to draw firm conclusions regarding clinical effectiveness. While both spatially bound and general mechanisms are partially understood, further work, particularly on molecular and imaging mechanisms, is needed. The recommended future research questions outlined here may help translate taVNS into broader clinical use. Prerequisites are promising: taVNS is cost-effective, non-invasive, and can be delivered during daily activities. Future studies should focus on technical advances and sharpened indications, and then embed taVNS into multimodal treatment concepts (e.g., combined with cognitive training) to maximize patient benefit in cognitive impairment associated with neuropsychiatric disease.

## Data Availability

The original contributions presented in the study are included in the article/Supplementary material, further inquiries can be directed to the corresponding authors.
