# Supplementary material for: Transcutaneous auricular vagus nerve stimulation may improve cognitive deficits in neuropsychiatric diseases—a systematic review
Source: Front Aging Neurosci. 2026 Feb 4;18:1735787. doi: 10.3389/fnagi.2026.1735787 (PMC12913560; doi:10.3389/fnagi.2026.1735787)
Supplement: Supplementary file 1 [file Table_1.docx]

**Supplementary table 1. Adverse events and safety reporting across included taVNS studies**

| ****Study**** | ****AE assessment reported**** | ****Adverse events described**** | ****Serious AEs**** | ****Summary comment**** |
| --- | --- | --- | --- | --- |
| ****Pan et al., 2024**** | Not reported | Not reported | Not reported | No mention of AEs |
| ****Uehara et al., 2022**** | Yes – hospital records | None; no AEs in active or sham group | No | taVNS safe and well tolerated; no local or systemic AEs. |
| ****Corrêa et al., 2022**** | Not reported | Not reported | Not reported | No mention of AEs |
| ****Marano et al., 2022**** | Not reported | Not reported | Not reported | No mention of AEs |
| ****Wang et al., 2022b**** | Yes – patient diary & phone follow-up | 1 patient (pre-existing ear injury): mild toothache, sore throat, tinnitus (resolved); no other AEs | No | taVNS safe and well tolerated; only one transient mild AE; no SAEs. |
| ****Evensen et al., 2022**** | Yes – UKU side-effect rating scale, SUS | Mild/moderate local effects (itching 13 %, pain 8 %, sensory 9.7 %); ↑ sleep length & itching; device handling issues | No | taVNS feasible and well tolerated; side effects mostly local and mild. |
| ****Lench et al., 2023**** | Yes – vitals, UPDRS-III, C-SSRS | Mild AEs (taVNS 33 %, sham 20 %): sleep disturbance, lightheadedness, fatigue, nausea, tinnitus; none severe | No | taVNS well tolerated; only transient mild AEs, no SAEs. |
| ****Mertens et al., 2022**** | Not reported | Not reported | Not reported | No mention of AEs |
| ****Weber et al., 2021**** | Yes – subjective reporting during stimulation | None; no subjective or physiological effects vs sham | No | taVNS well tolerated; no adverse or serious events observed. |
| ****Oehrn et al., 2022**** | Not reported | Not reported | Not reported | No mention of AEs |
| ****Stefan et al., 2012b**** | Yes – patient questionnaires | Hoarseness, headache, obstipation; 3 drop-outs due to minor AEs | No | Long-term taVNS safe and well tolerated; no serious or lasting effects. |
| ****Trevizol et al., 2016**** | Yes – clinical follow-up (Day 0, 10, 45) | Mild paresthesia (all), sleepiness (10), headache (6), nausea (4); none at follow-up | No | taVNS safe and well tolerated; only transient mild AEs. |
| ****von Wrede et al., 2021b**** | Yes – clinical check-up + AEP questionnaire | None; no local or systemic AEs; good device tolerability | No | Short-term taVNS safe and well tolerated; no AEs occurred. |
| ****Yang et al., 2023a**** | Yes – patient reports, vitals, ECG | Mild AEs (11 % active, 19 % sham): headache, insomnia, flu-like symptoms, local erythema; 1 transient bradycardia | No | taVNS well tolerated; mild, transient AEs only; no serious events. |
| ****Zheng et al., 2024**** | Not reported | Not reported | Not reported | No mention of AEs |

**Abbreviations:** AE = adverse event; AEP = Adverse Events Profile; C-SSRS = Columbia–Suicide Severity Rating Scale; ECG = electrocardiogram; SAE = serious adverse event; SUS = System Usability Scale; taVNS = transcutaneous auricular vagus nerve stimulation; UPDRS = Unified Parkinson’s Disease Rating Scale.
